# Supplementary material for: Conductive porous vanadium nitride/graphene composite as chemical anchor of polysulfides for lithium-sulfur batteries
Source: Nat Commun. 2017 Mar 3;8:14627. doi: 10.1038/ncomms14627 (PMC5337987; doi:10.1038/ncomms14627)
Supplement: Supplementary Information — Supplementary Figures 1-10, Supplementary Table 1, Supplementary Note 1 and Supplementary References [file ncomms14627-s1.pdf]

Supplementary Figures

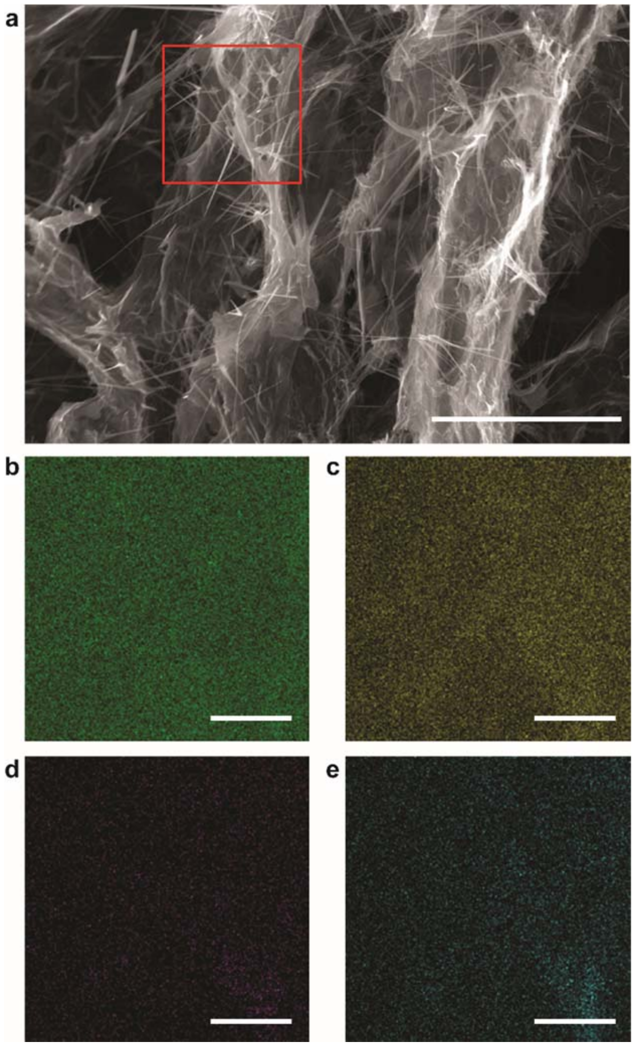

Supplementary Figure 1 SEM and EDX mapping characterizations of materials. (**a**) SEM image of the VN/G composite, and EDX elemental maps of (**b**) carbon, (**c**) vanadium, (**d**) nitrogen and (**e**) oxygen in the red solid line area, respectively. Scale bars, 5μm (**a**), 1 μm (**b-e**).

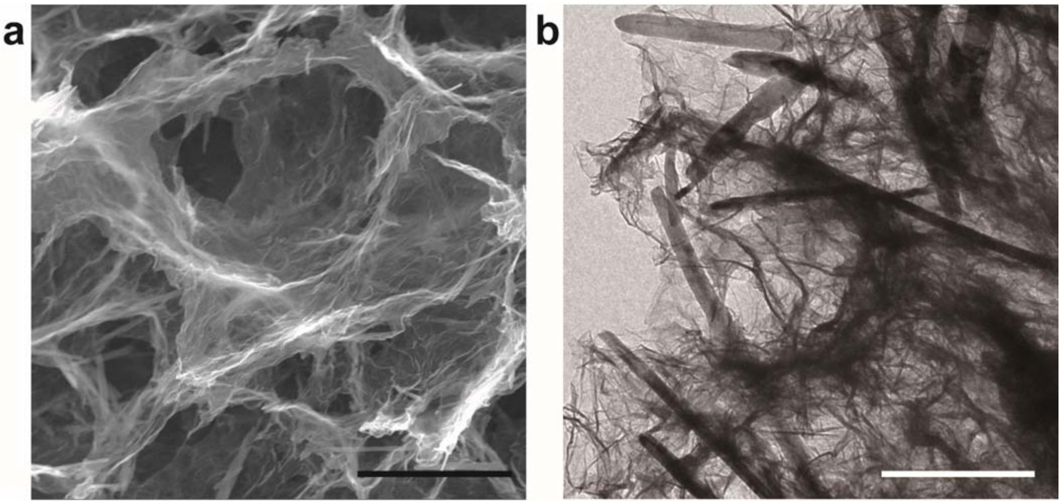

Supplementary Figure 2 Characterization of the VO_x/G composite. (a) SEM image and (b) TEM image of the VOx/G composite before annealing in NH_3. Scale bars, 2μm (a), 500nm (b).

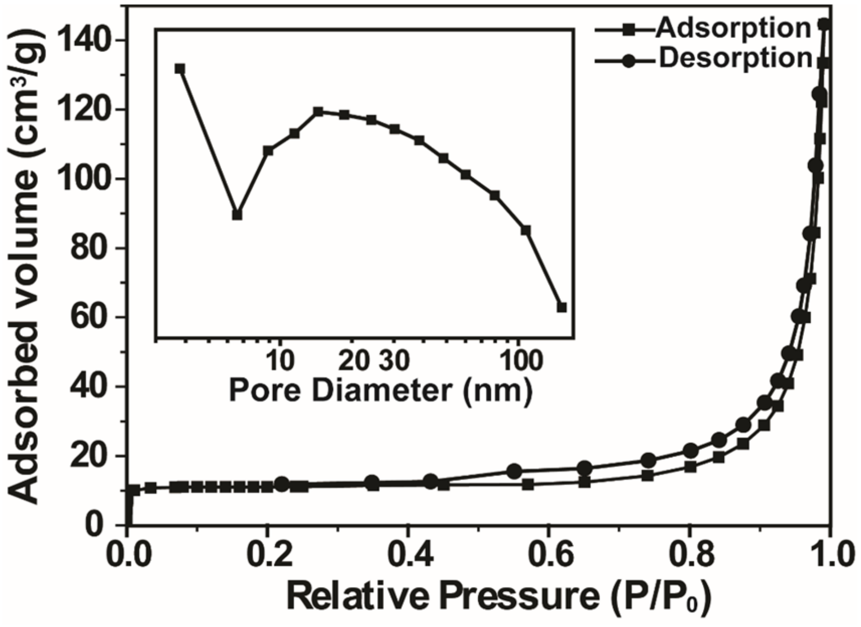

Supplementary Figure 3. Nitrogen adsorption-desorption isotherm of the VN/G composite. Inset: the pore size distribution obtained using the BJH method.

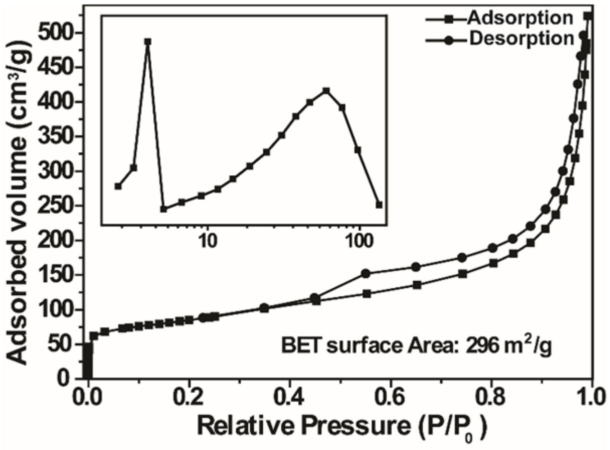

Supplementary Figure 4. Nitrogen adsorption-desorption isotherm of the RGO. Inset: the pore size distribution obtained using the BJH method.

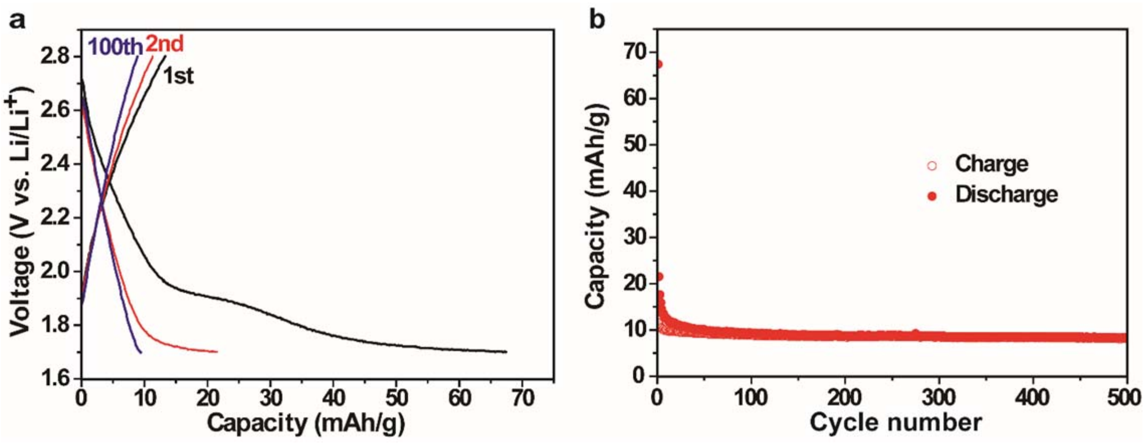

Supplementary Figure 5 Electrochemical performances of the VN/G composite without Li_2S_6 catholyte. (**a**) Charge-discharge profiles and (**b**) Cycling performance of the VN/G composite without Li_2S_6 catholyte at a current density of 335 mA g^{-1} in the voltage range 2.8-1.7 V. The pure VN/G composite shows a capacity of < 10 mAh g^{-1} at a current density of 335 mA g^{-1} in the voltage range 2.8-1.7 V.

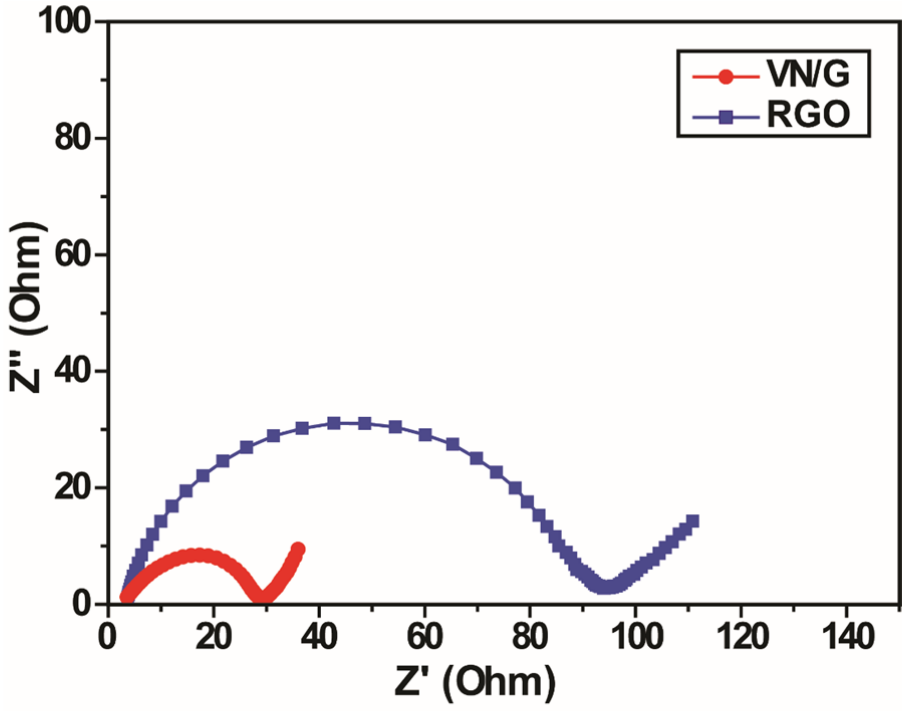

Supplementary Figure 6. Comparison of the electrochemical impedance spectra of the VN/G and RGO cathodes before cycling. The data was recorded from 10 kHz to 100 MHz at open circuit voltage at room temperature.

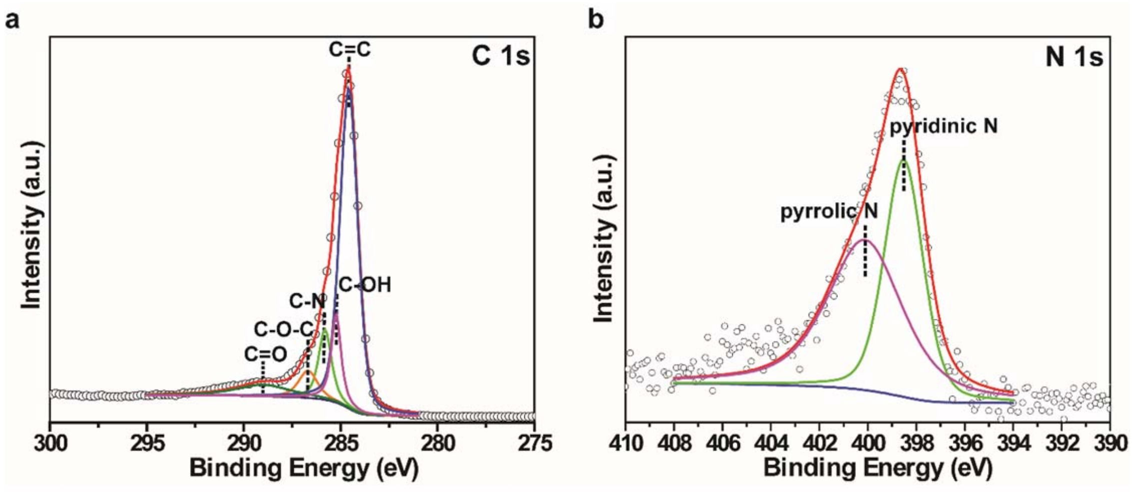

Supplementary Figure 7 XPS spectra of the RGO. (**a**) High-resolution C1s and (**b**) N1s XPS spectra acquired from the RGO.

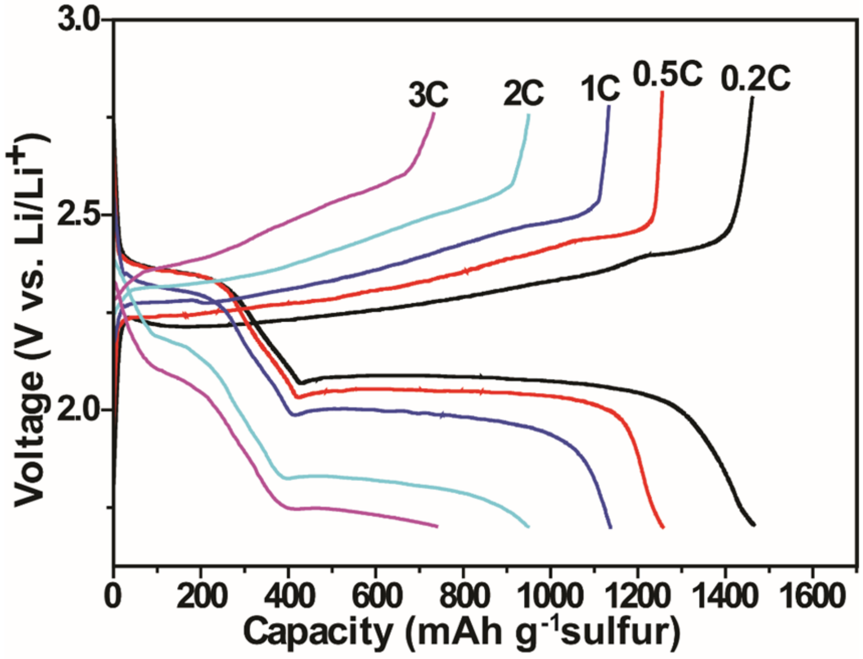

Supplementary Figure 8. Galvanostatic charge-discharge profiles of the VN/G cathode at different rates.

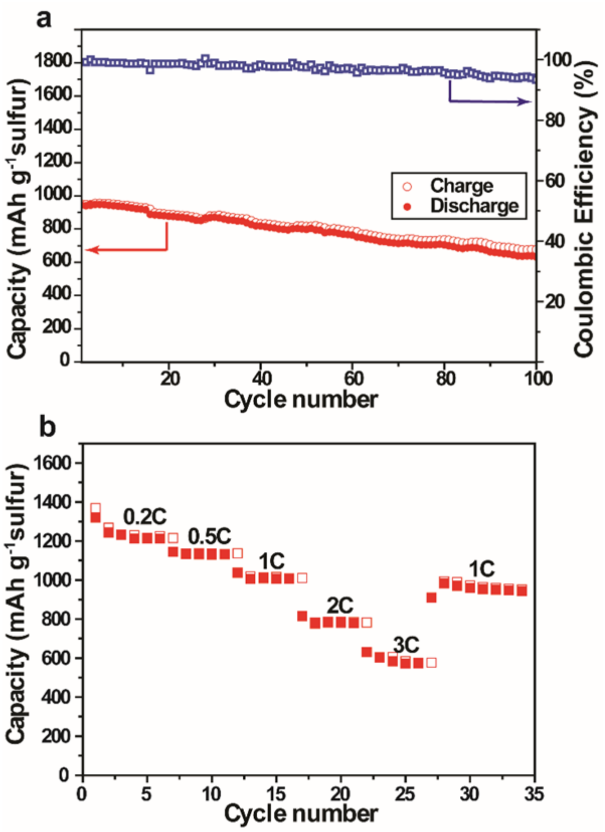

Supplementary Figure 9 Electrochemical performances of the VO_x/G cathode. (**a**) Cycling stability at 1C and (**b**) rate performance of the VO_x/G cathode.

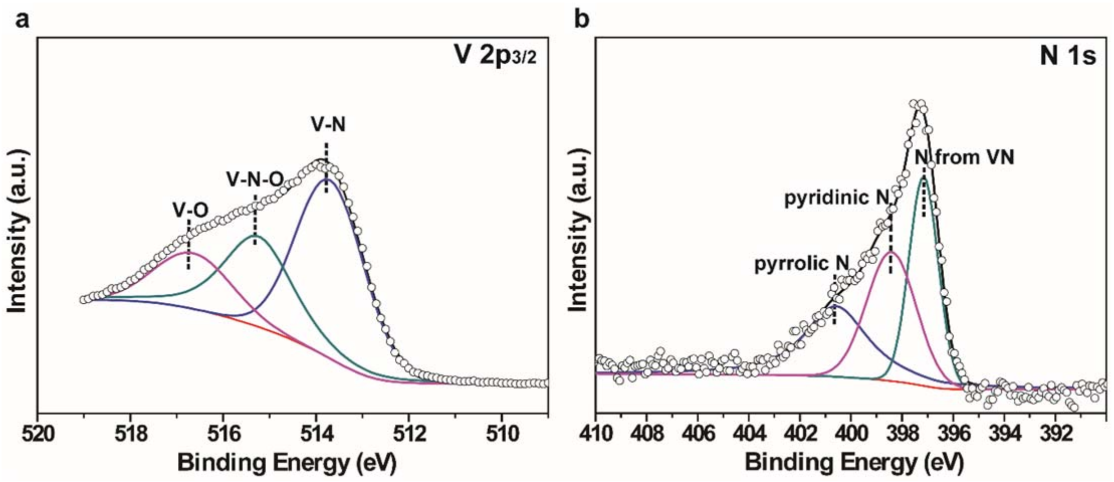

Supplementary Figure 10 XPS spectra of the VN/G composite. (**a**) High-resolution $V2p_{3/2}$ and (**b**) N1s XPS spectra acquired from the VN/G composite.

Supplementary Table

Supplementary Table 1 Electrical conductivity of different metal nitrides at room temperature[1].

Materials	VN	TiN	Mo_2N	WN	Ni_3N
Conductivity ($\times 10^6$ S m^{-1})	1.17	4.0	5.05	11.1	0.36

Supplementary Note

Supplementary Note 1. Theoretical calculation Methods

DFT calculations were performed using the projector augmented wave method [2,3] and a plane-wave (PW) basis set as implemented in the Vienna *ab-initio* simulation package[4]. The Perdew-Burke-Ernzerhof functional[5] for the exchange-correlation term was used for all calculations. The energy cutoff for the PW basis set was set to be 400 eV. For the structural relaxations and energy calculations, vdW interactions were incorporated by the optB88 exchange functional[6,7], and this proved to be very important to accurately evaluate the interactions between the S-containing clusters and pristine graphene[8]. In order to calculate the binding energy between Li_2S_6 and VN, a slab model of a 3×3×1 supercell with four atomic layers was constructed to simulate the (200) surface of VN substrate based on HRTEM observations. One edge C atom was substituted by the N atom in a large poly-aromatic hydrocarbon (PAH) molecule of $C_{96}H_{24}$, resulting in a N-doped PAH molecule (C $C_{95}N_1H_{23}$) to represent N-doped graphene (N-G) with a pyridinic N atom in a 30×30×20 $Å^3$ supercell, the size of this PAH molecule was confirmed to be large enough to accurately evaluate the interactions between S-containing clusters lithium polysulfides and graphene[9]. A Γ-centered k-mesh (3×3×1) and only the Γ point was used to sample the first Brillouin zone of the VN slab model and the PAH supercell, respectively, for the structural relaxation and energy calculations. Except for the bottom two atomic layers of the VN slab, all atoms were allowed to be fully relaxed while keeping the supercell boxes unchanged until the residual force per atom decreases to less than 0.01 eV $Å^{-1}$. The binding energies between Li_2S_6 and VN (N-G) are defined by equation (1):

$$E_b = E_T - E_S - E_{Li_2S_6} \qquad (1)$$

where E_{Li2S6}, E_S, and E_T are, respectively, the total energies of an isolated Li_2S_6 cluster, substrate (N-doped graphene or VN), and a composite system of the substrate with Li_2S_6.

Supplementary References

1. Oyama, S. T. *The chemistry of transition metal carbides and nitrides* Ch.1, (Chapman & Hall Press, 1996).
2. Blöchl, P. E. Projector augmented-wave method. *Phys. Rev. B* **50**, 17953-17979 (1994).
3. Kresse, G. & Joubert, D. From ultrasoft pseudopotentials to the projector augmented-wave method. *Phys. Rev. B* **59**, 1758-1775 (1999).
4. Kresse, G. & Furthmüller, J. Efficient iterative schemes for ab initio total-energy calculations using a plane-wave basis set. *Phys. Rev. B* **54**, 11169-11186 (1996).
5. Perdew, J. P., Burke, K. & Ernzerhof, M. Generalized gradient approximation made simple. *Phys. Rev. Lett.* **77**, 3865-3868 (1996).
6. Klimeš, J., Bowler, D. R. & Michaelides, A. Van der Waals density functionals applied to solids. *Phys. Rev. B* **83**, 195131 (2011).
7. Klimeš, J., Bowler, D. R. & Michaelides, A. Chemical accuracy for the van der Waals density functional. *J. Phys.: Condens. Matter. Phys.* **22**, 022201 (2010).
8. Zhang, Q. F. *et al.* Understanding the anchoring effect of two-dimensional layered materials for lithium-sulfur batteries. *Nano Lett.* **15**, 3780-3786 (2015).
9. Yin, L. C., *et al.* Understanding the interactions between lithium polysulfides and N-doped graphene using density functional theory calculations. *Nano Energy* **25**, 203-210 (2016).
